# Systems serology-based comparison of antibody effector functions induced by adjuvanted vaccines to guide vaccine design

**DOI:** 10.1038/s41541-023-00613-1

**Published:** 2023-03-08

**Authors:** Carolin Loos, Margherita Coccia, Arnaud M. Didierlaurent, Ahmed Essaghir, Jonathan K. Fallon, Douglas Lauffenburger, Corinne Luedemann, Ashlin Michell, Robbert van der Most, Alex Lee Zhu, Galit Alter, Wivine Burny

**Affiliations:** 1 The Ragon Institute of MGH, MIT and Harvard, Cambridge, MA USA; 2 GSK, Rixensart, Belgium; 3 Virology and Immunology Program, University of Duisburg-Essen, Essen, Germany; 4 Present Address: Center of Vaccinology, University of Geneva, Geneva, Switzerland

**Keywords:** Antibodies, Biomarkers

## Abstract

The mechanisms by which antibodies confer protection vary across vaccines, ranging from simple neutralization to functions requiring innate immune recruitment via Fc-dependent mechanisms. The role of adjuvants in shaping the maturation of antibody-effector functions remains under investigated. Using systems serology, we compared adjuvants in licensed vaccines (AS01_B_/AS01_E_/AS03/AS04/Alum) combined with a model antigen. Antigen-naive adults received two adjuvanted immunizations followed by late revaccination with fractional-dosed non-adjuvanted antigen (NCT00805389). A dichotomy in response quantities/qualities emerged post-dose 2 between AS01_B_/AS01_E_/AS03 and AS04/Alum, based on four features related to immunoglobulin titers or Fc-effector functions. AS01_B/E_ and AS03 induced similar robust responses that were boosted upon revaccination, suggesting that memory B-cell programming by the adjuvanted vaccinations dictated responses post non-adjuvanted boost. AS04 and Alum induced weaker responses, that were dissimilar with enhanced functionalities for AS04. Distinct adjuvant classes can be leveraged to tune antibody-effector functions, where selective vaccine formulation using adjuvants with different immunological properties may direct antigen-specific antibody functions.

## Introduction

A durable, functional antibody response represents the primary immune correlate of protection for most licensed vaccines^1,2^. Yet, single-function measurements of the humoral immune response, such as binding immunoglobulin (Ig)G or neutralizing antibody (NAb) titers, often incompletely or inconsistently correlate with protection^2,3^. The importance of additional functions of antibodies in protection from infection has progressively emerged across several diseases. Specifically, the ability of antibodies to recruit innate immune effector functions is critical in protection against influenza^4,5^, anthrax^6^, malaria^7^, human immunodeficiency virus (HIV)^8^, and many more infections^9–11^. Indeed, it is now widely accepted that antibodies of different isotypes, subclasses, and Fc-glycosylation profiles can interact with Fc receptors or complement with different affinities. By driving distinct extra-neutralizing functions in the immune response^3,12^, such interactions thus connect the adaptive and innate arms of the immune system. However, it remains unclear how these antibody properties and functions are tuned immunologically, and whether they may be rationally harnessed via vaccination to improve pathogen control and clearance. Yet, both functional and biophysical Fc properties can be surveyed by the high-throughput assays and computational analyses used in systems serology approaches^13^. The latter provide complementary insights to commonly assessed Fab properties such as affinity, repertoire breadth, and neutralization potency.

Challenges faced by modern vaccine development include variable immunocompetence and priming statuses in the target populations. This is particularly relevant for vaccines for older adults, infants, and the immunosuppressed^1,2,14^. Moreover, the vaccination context dictates a focus on instantly provided protection, e.g., for traveler or pandemic vaccines, and/or on long-term effectiveness, such as for malaria or tuberculosis vaccines in disease-endemic regions^15–17^. These considerations provide the impetus to develop novel vaccine strategies tailored to the vaccine indication. Induction of robust and durable antibody responses to protein antigens depends on innate immune stimulation, and vaccines using antigens inherently lacking immune stimulatory properties can be improved by an adjuvant^1,18^. Beyond Alum, several novel adjuvants and adjuvant combinations, or “Adjuvant Systems”^19^, have been incorporated into licensed vaccines or pandemic candidate vaccines. For example, Adjuvant System (AS)01_B_ (liposome containing the Toll-like receptor [TLR]4 agonist MPL, and the saponin QS-21) is included in the herpes zoster vaccine, AS01_E_ (half the MPL and QS-21 amounts compared to AS01_B_) is a component of the *Plasmodium falciparum* malaria vaccine RTS,S, undergoing implementation studies, and AS03 (oil-in-water emulsion + α-tocopherol) has been incorporated into (pre)pandemic vaccines against influenza or SARS-CoV-2^1,16,18–21^. In addition, AS04 (MPL adsorbed onto Alum in the form of AlPO_4_) is part of recombinant human papillomavirus types 16/18 (HPV-16/18) and hepatitis B virus (HBV) vaccines^19^. Considering their abilities to boost antigen uptake/processing, costimulatory molecules, and T-cell activation^1^, adjuvants have historically been evaluated based largely on the magnitude of the cell-mediated and humoral immune responses they induce. However, nonhuman primate (NHP) data suggest that adjuvants can tune antibody-effector functions as well^22^. Moreover, activation of Fc-functional features was detected in human vaccinees who received RTS,S (malaria antigen fused to HBV surface antigen [HBsAg]) formulated in AS01_B_^7^. Due to the lack of a control group in that study, however, the mechanisms underlying adjuvant-induced modulation of effector functions remain unclear, for AS01_B_ as well as for the other adjuvants used in licensed vaccines.

Here, we aimed to directly contrast the functional humoral consequences of AS01_B_, AS01_E_, AS03, AS04, or Alum (Al(OH)_3_) for vaccine-induced immunity. We analyzed samples from a clinical vaccine trial in which HBV-naive participants received two doses, 1 month apart, of HBsAg formulated with one of these adjuvants^23–26^. One year later, they received a fractional-dose antigenic challenge (non-adjuvanted HBsAg) to probe the adjuvants’ effects on immune memory^26^. Previously, these distinct adjuvants were compared focusing on the innate and adaptive responses after both adjuvanted doses, and on antibody avidity levels up to one month post-antigenic challenge^23–26^. When comparing AS01_B_ and AS01_E_, we demonstrated many similarities that extended to the oil-in-water adjuvant system AS03, but we also observed differences in their innate immune activation profiles or adaptive immune responses. While the difference between AS01_E_ and AS01_B_ may be partially explained by the difference in the MPL and QS-21 doses of these adjuvants, the specifics of the behavior of these constituents in the liposomal formulations could follow a non-linear dose-effect relationship, for example in the effects on the molecular and cellular MPL/QS-21 synergy^27^ on the immune response. Comparing the five adjuvant systems in these collective post-hoc analyses creates an important dynamic range and consistency with this previously published work, allowing us to interlink the different immune features to better understand the mode of action of these adjuvanted vaccines. In the current report, we used a systems serology approach to evaluate the Fc-biophysical and functional properties of the HBsAg-specific antibodies across the three doses. This allowed us to compare these features both across adjuvants, and between adjuvanted and non-adjuvanted doses. Differences in antibody features appeared after the second dose, marked by the emergence of distinct adjuvant clusters: a robust Fc-profile induced by AS01_B_/AS01_E_/AS03, a moderate functional profile induced by AS04, and a weak, more narrow functional profile induced by Alum. Moreover, these functional differences were recalled and differently modified across groups after unadjuvanted antigenic challenge, as seen for antibody avidity^26^, highlighting functional programming in the memory response. The data can guide the rational selection of adjuvants and immunization schedules for future subunit vaccines.

## Results

### Generating a comprehensive humoral immune profile per adjuvant group

Using systems serology^13^, we deeply profiled the biophysical and functional characteristics of the humoral immune response measured in serum, as induced by HBsAg vaccines formulated with AS01_B_, AS01_E_, AS03, AS04, or Alum. Using an Fc-binding protein array, HBsAg-specific total IgG, IgG_1-4_, IgM, and IgA_1-2_ levels were interrogated, as were the abilities of HBsAg-specific antibodies to bind to activating or inhibitory Fc gamma receptors (FcGRs: FcGRIIA, FcGRIIB, FcGRIIIA, and FcGRIIIB), or to FcRn, FcAR, or complement C1q. In addition, we assessed the capacity of HBsAg-specific antibodies to drive antibody-dependent complement deposition (ADCD) or primary natural killer-cell activation (ADNKA), or antibody-dependent phagocytosis by either THP-1 cells (ADCP), primary neutrophils (ADNP), or MoDCs, i.e., monocyte-derived dendritic cells (ADDCP). A total of 24 antibody features were captured for each subject, at days 30 (1 month post-dose 1), 60 (1 month post-dose 2), 360 (1 year post-dose 1), and 390 (1 month post-antigenic challenge).

**Fig. 1:**
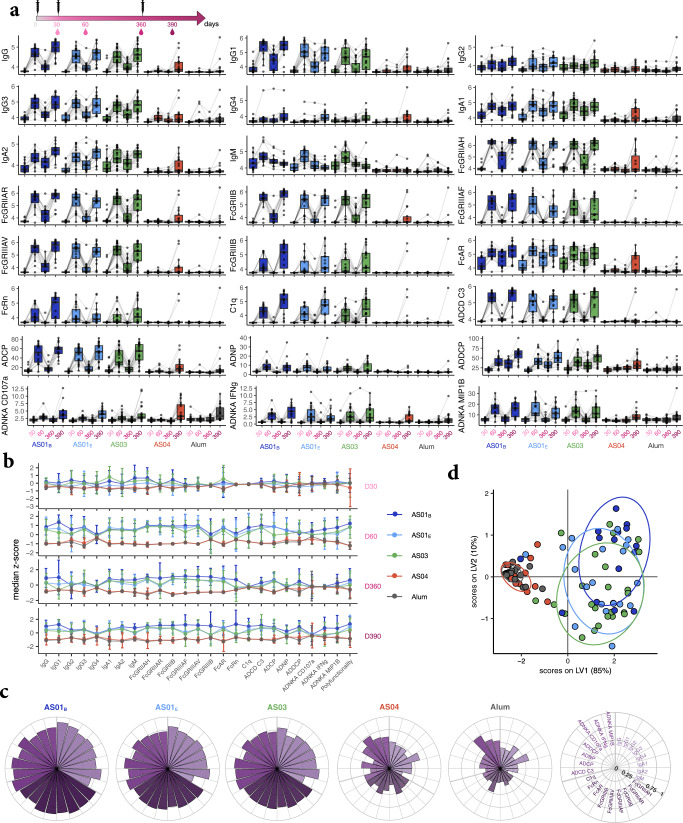
Adjuvants shape vaccine-induced functional antibody responses. **a** The boxplots (representing medians, interquartile ranges [IQRs], minima, and maxima) show the antibody features for each vaccine adjuvant group. Groups received HBsAg adjuvanted with AS01_B_, AS01_E_, AS03, AS04, or Alum. Samples were profiled at day 30, 60, 360, and 390. Individuals were vaccinated at day 0, with an adjuvanted boost at day 30 and a non-adjuvanted, fractional-dose antigenic challenge at day 360. Measurements are provided as: log_10_ MFI (mean fluorescence intensity), for the isotypes/subclasses/FcR-binding levels and C1q; as phagocytosis score, for antibody-dependent cellular phagocytosis (ADCP), antibody-dependent neutrophil phagocytosis (ADNP), and antibody-dependent dendritic cell phagocytosis (ADDCP); and as percentage of cells that are positive for each activation marker (CD107a, IFN-γ, MIP-1β), for antibody-dependent NK-cell activation (ADNKA). **b** Each row shows the median values and IQRs for the antibody features at one time point. The measurements were z-scored for each time point and across all adjuvant groups. **c** The polar plots depict the mean percentile of each antibody feature for each adjuvant group at day 390. Percentile rank scores were determined for each antibody feature across all individuals. **d** A partial least square discriminant analysis (PLS-DA) model was generated based on LASSO-selected features from all time points and area under the curve (AUC). Each dot represents a vaccinated subject in the PLS-scores plot. Ellipses show 75% confidence regions assuming a multivariate *t* distribution.

The univariate analysis highlighted the robust responses to HBsAg in the presence of AS01_B_, AS01_E_, and AS03 following the second vaccine dose, with interindividual heterogeneity illustrated by the boxplots (Fig. 1a). Most immunized subjects in these three groups raised detectable levels of all isotypes and subclasses at day 60. In addition, at that same time point the vaccine-induced antibodies robustly engaged Fc receptors and induced multiple Fc-effector functions, including complement deposition, NK-cell activation, and phagocytosis by multiple innate cell types. Antibody levels subsequently waned between days 60 and 360, but were still readily detectable at day 360 in most subjects in these three groups. In addition, and as expected, we found that the antigenic challenge increased HBsAg-specific IgG but not IgM levels (Fig. 1a), demonstrating class switching.

As previously shown for these subjects^25,26^, AS01_B_, AS01_E_, and AS03 induced overall higher (Ig) antibody responses compared to AS04 and Alum, across all four time points. Interestingly, though some intergroup differences were already observed after the first dose (e.g., for IgA_1,2_, IgM, FcAR), the levels for most antibody features displayed only slight differences across the five adjuvant groups, in spite of the different titers (Fig. 1b and Supplemental Table 1). Conversely, after the adjuvanted boost, large differences were noted between the AS01_B_, AS01_E_, or AS03 groups versus the AS04 or Alum groups (Fig. 1b, c [day 390], Supplemental Fig. 1 [other time points]). Most critically, this functional profile in the former three groups persisted to day 360 (Fig. 1a). After the second dose, responses induced by AS04 and Alum were significantly lower and contained higher relative proportions of the less effective IgG_4_ subclass as compared to AS01 or AS03. Additionally, at day 390 the responses for some features (e.g., IgA1, FcAR, FcGRIIIAH, ADNKA/CD107a) tended to be more heterogeneously induced across the participants receiving AS04 versus those receiving AS01 or AS03. Overall, at a univariate level, AS01_B_, AS01_E_, and AS03 showed enhanced quantitative and qualitative alterations in the humoral immune response, whereas the responses induced by AS04 and Alum lagged but reached for some parameters comparable levels in a subset of vaccinees, mostly after the antigenic challenge.

### Dissecting differences between the humoral immune responses to different adjuvants

Despite the differences in antibody quality across several groups, it was not clear whether a set of features could resolve all five adjuvant profiles. To probe whether a multivariate profile could discriminate qualitative differences across adjuvants, we employed an unbiased machine learning approach to compare the multifaceted humoral profiles induced in each adjuvant group. In addition to the 24 antibody measurements per time point, we also summarized the features by including both a polyfunctionality score for each subject (calculated as the number of functional readouts exceeding the median across all subjects/groups) and the total area under the curve (AUC; as a proxy for time) for each readout. Using a least absolute shrinkage and selection operator (LASSO), a set of five discriminating features (i.e., days 60 and 360 FcGRIIAH, day 60 FcAR, IgA_1_, and IgG_1_ AUCs) were selected (Supplemental Fig. 2a). These discriminatory features were all enriched in the AS01_B_, AS01_E_, and AS03 groups. Moreover, subsequent partial least squares discriminant analysis (PLS-DA) on the selected features in a tenfold cross-validation framework revealed that these features were able to separate the adjuvant profiles into two clusters: one composed of AS01_B_/AS01_E_/AS03 HBsAg vaccinees, and a second, non-overlapping cluster of AS04/Alum HBsAg vaccinees (Fig. 1d). Thus, while slight differences were noted across all adjuvant arms, the strongest divergence emerged across two adjuvant clusters, which was driven by both quantitative and qualitative differences in the humoral immune response.

### Evaluating the correlations between antibody features

**Fig. 2:**
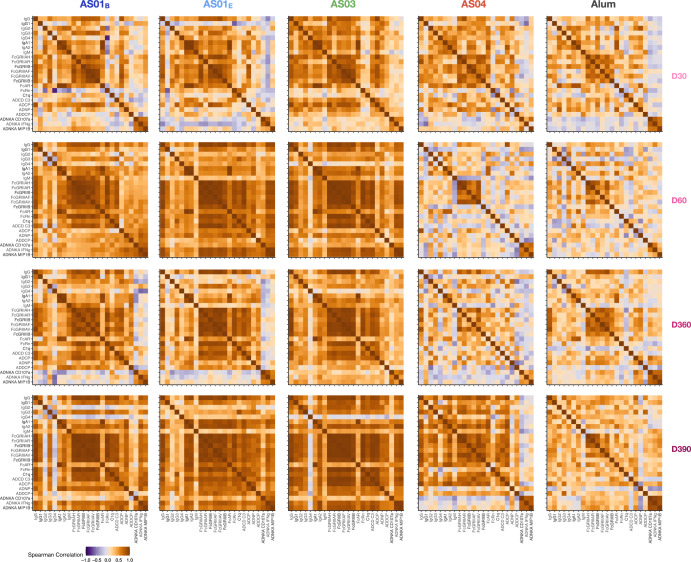
Differences in correlation structures. Correlation heatmaps were generated for each adjuvant (column) at each time point (row), using Spearman rank correlations. For each adjuvant/time point, all correlations between humoral features were calculated, with orange indicating positive and purple indicating negative correlations. ADNP antibody-dependent neutrophil phagocytosis, ADCP antibody-dependent phagocytosis by THP-1 cells, ADCD antibody-dependent complement deposition, ADNKA antibody-dependent natural-killer cell activation. ADNP antibody-dependent phagocytosis by primary neutrophils, ADDCP antibody-dependent phagocytosis by monocyte-derived dendritic cells.

Both the multivariate and univariate profiles pointed to similarities in antibody quantity and quality within the clusters formed by AS01_B_/AS01_E_/AS03 or AS04/Alum (Fig. 1). However, whether the overall architecture and coordination of the humoral response were still similar within each individual adjuvant group remained unclear. Thus, we next aimed to probe the overall coordination of the humoral responses across subjects within each group. Spearman rank correlation coefficients for every pair of features highlighted the different correlation patterns induced by the adjuvants by time point (Fig. 2). Overall, the AS01_B_, AS01_E_, and AS03 groups each showed stronger correlations between antibody features than the AS04 and Alum groups. For AS01_B_, AS01_E_, and AS03, coordination between Fc-receptor (FcR)-binding levels and ADCP activity was already observable at day 30, whereas strong correlations between FcR engagement and other functional features only arose after either the second adjuvanted dose (day 60) or the antigenic challenge (day 390). Conversely, for the AS04 arm strong correlations were only observed at day 60 (between different FcR-binding antibody levels) or day 390 (between antibody titers, FcR-binding levels, and some functional features). For the Alum arm, only some pairs of FcR-binding antibody features displayed strong correlations across time points. These differences pointed to further qualitative differences between these adjuvants.

### Identifying the most discriminating features between the two adjuvant clusters

**Fig. 3:**
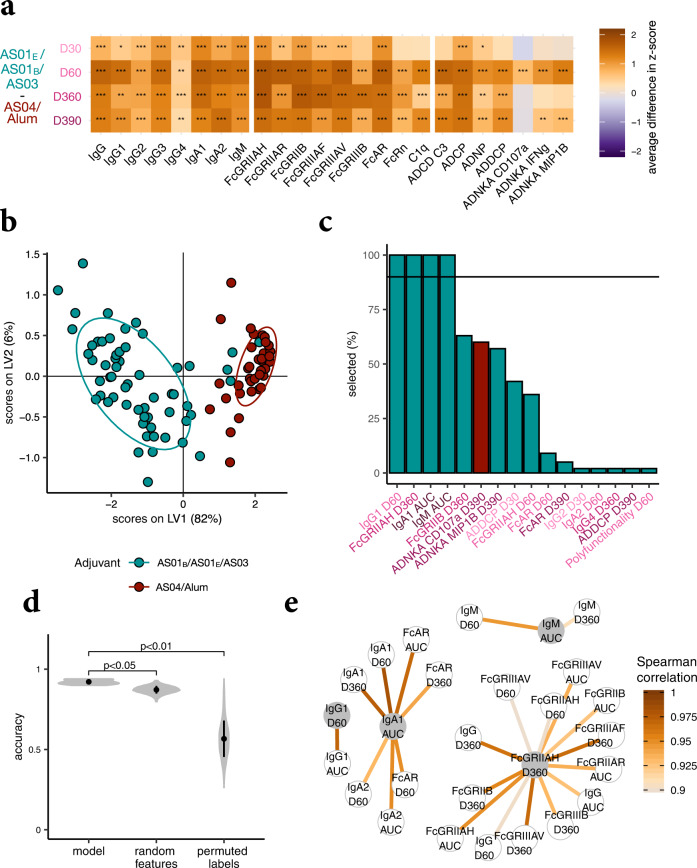
Dissecting differences between adjuvant clusters. **a** The heatmap shows differences in the antibody features between the merged AS01_B_/AS01_E_/AS03 and AS04/Alum-adjuvanted vaccine groups over time. Orange tiles indicate that the feature is on average higher in subjects receiving the HBsAg vaccines containing AS01_B,_ AS01_E_, or AS03, while purple tiles show enrichment in participants receiving HBsAg with AS04 or Alum. Significances were assessed using Mann–Whitney *U* tests and corrected for multiple testing using the Benjamini–Hochberg procedure. Asterisks indicate adjusted *P* values with *P < 0.05, **P < 0.01, ***P < 0.001. **b** A PLS-DA model was built based on features selected from all time points and the areas under the curves (AUCs) for the clusters AS01_B_/AS01_E_/AS03 and AS04/Alum. **c** The bar graph depicts how often antibody features were selected by repeated LASSO-based selection. The color indicates the adjuvant cluster in which the feature is enriched. The horizontal line shows the threshold of how often a feature needs to be chosen overall in order to be selected for the final set of minimal features. **d** The modeling approach was validated using permutation tests, for which the performance measured as classification accuracy of the actual model (using the four selected features shown in panel **c**) is compared to control models in a cross-validation framework. For “random features”, a fold-specific set of features of the same size as obtained by the LASSO-selection were chosen to train the model, and for “permuted labels” the modeling approaches were applied to shuffled group labels. The violin plots show the distribution of classification accuracies, for 10 repetitions and 100 permutations for the control models, and the *P* values indicate the median over the 10 repetitions of the exact *P* values obtained by permutation testing. **e** A co-correlate network was constructed using Spearman rank correlations. Only correlations with |r| > 0.9 to at least one of the selected features, which are highlighted in gray, are shown.

The preceding analyses detected striking quantitative differences in the antibody responses induced by the AS01_B_/AS01_E_/AS03 cluster versus the AS04/Alum cluster. Next, we aimed to identify the features that differed most across the groups, by defining the strongest functional disparities across the two adjuvant clusters. A heatmap presentation of the differences between clusters revealed strongly diverging antibody features (Fig. 3a). To avoid overfitting, a LASSO-based selection was then performed to identify the minimal features that differed most across the two adjuvant clusters, followed by PLS-DA to classify and visualize the data. Only four antibody features sufficed to completely split the two clusters, with a cross-validation accuracy of 91.41% (Fig. 3b). These four features were selected in each of 100 repetitions of the LASSO-based feature selection. The features included vaccine-induced IgG_1_ titers at day 60, FcGRIIAH-binding antibody levels at day 360, and AUCs of the IgA_1_ and IgM titers (Fig. 3c). Moreover, this model significantly outperformed models based on random features and permuted data (*P* < 0.05 and *P* < 0.01, respectively, Fig. 3d). The high accuracy of the random feature model was likely due to the high correlation of individual features. Thus, the features selected by this model highlighted discrete priming and longitudinal titer differences between the clusters (i.e., day 60 IgG_1_, AUCs of IgA_1_ or IgM). They also underscored the importance of qualitative differences in Fc-receptor binding at boosting time points (day 360 FcGRIIAH) in differentiating these clusters. In short, prime, boost, and longitudinal differences in post-vaccination titers together, rather than single-antibody features, highlighted the difference between adjuvant clusters.

Given the highly correlated nature of the humoral immune response, we next aimed at gaining additional mechanistic insights into linked humoral changes that differed across the adjuvant clusters. Specifically, the co-correlates (Spearman correlation >0.9) of the four LASSO-selected features were examined (Fig. 3e). The FcGRIIAH-binding antibody levels at day 360 were highly correlated with other FcR-binding antibody features or IgG titers at different time points across the subjects, marking superior Fc-effector function in the AS01_B_/AS01_E_/AS03 adjuvant cluster over time. Similarly, the AUC of IgA_1_ levels was highly correlated with other IgA_1_, IgA_2_, and FcAR-binding antibody levels, showing enhanced overall IgA-induced immunity in the cluster. The other two LASSO-selected features were more unique: HBsAg-specific IgG_1_ levels at day 60 only correlated strongly with the AUC of IgG_1_ levels, while the AUC of HBsAg-specific IgM levels only correlated well with IgM levels at day 60. Importantly, analyzing these correlation networks further highlights the specificity of the four-feature predictive model, as the 24 features measured were not readily interchangeable, and thus not selectively altered across the adjuvant groups. Overall, while both adjuvant clusters induced several antibody-effector functions, the AS01_B_/AS01_E_/AS03 cluster induced more robust and durable FcR-engaging and IgA-biased responses. The AS04/Alum cluster also induced antibody-effector functions, but in a more tempered manner, and marked by lower IgA immunity.

### Similar profiles of AS01_B_, AS01_E_, and AS03

**Fig. 4:**
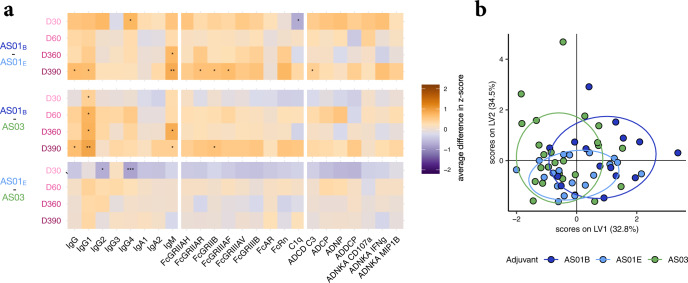
Similarity in functional antibody responses across AS01_B_-, AS01_E_-, and AS03- adjuvanted vaccine responses. **a** The heatmaps depict pairwise differences in antibody features between AS01_B_ versus AS01_E_ participant groups (top), AS01_B_ vs. AS03 participant groups (middle), and AS01_E_ vs. AS03 participant groups (bottom). Orange tiles indicate that the feature is higher in the first adjuvant group, while purple tiles show enrichment in the second adjuvant group. Significances were assessed using Mann–Whitney *U* tests, and asterisks indicate uncorrected *P* values with *P < 0.05, **P < 0.01, ***P < 0.001. **b** A PLS-DA model was built based on LASSO-selected features. The score plot shows high overlap between the high-response adjuvants AS01_B_, AS01_E_, and AS03, with the strongest overlap between AS01_E_ and AS03. The model only achieved a balanced accuracy of 0.23 and was not able to discriminate the adjuvants.

While AS01_B_, AS01_E_, and AS03 clustered together in the cross-adjuvant analysis, they are distinct with respect to the nature (AS01_B/E_ versus AS03) or concentration (MPL/QS-21 in AS01_B_ versus AS01_E_) of their constituents. Specific aspects of the formulation of these adjuvants are known to differentially affect the innate immune stimulation—e.g., as described for particulates, such as Alum, versus non-particulate adjuvants^28,29^. In turn, such differences can modify the adaptive immune responses, including Fc features. Thus, to next define whether these three adjuvants also induced distinct antibody profiles, multivariate analysis was performed for each pair of vaccine arms. Both qualitative and quantitative differences were observed. Individuals who received AS01_B_ displayed a higher average response for most subclasses, isotypes and FcR-binding features compared to those receiving AS01_E_ or AS03 (Fig. 4a), marking potential variation within this adjuvant cluster. However, the three vaccine arms induced similar antibody profiles that could not be robustly separated using LASSO- and PLS-DA-based multivariate analysis (Fig. 4b and Supplementary Fig. 2b), pointing to overall similar qualitative responses across the three adjuvants. This suggested a potential equivalence of these potent adjuvants with respect to the qualitative Fc response we explored in the context of HBsAg.

### Slight differences between profiles of AS04 and Alum

**Fig. 5:**
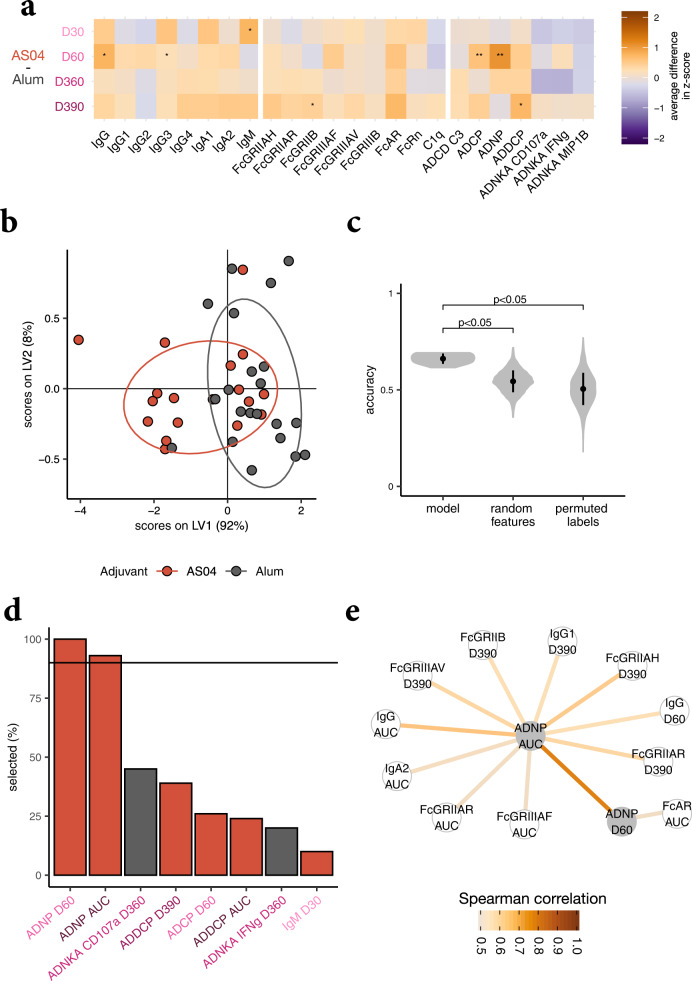
Slight difference in humoral profiles across responses induced by the AS04- or Alum-adjuvanted vaccines. **a** The heatmap shows differences in the antibody features between the AS04 or Alum-adjuvanted vaccine groups over time. Orange tiles indicate that the feature is on average higher in HBsAg/AS04-vaccinated individuals, while purple tiles show enrichment in HBsAg/Alum vaccinees. Significances were assessed using Mann–Whitney *U* tests, and asterisks indicate uncorrected *P* values with *P < 0.05, **P < 0.01, ***P < 0.001. **b** The PLS-DA score plot shows a slight separation between AS04 and Alum using ADNP at day 60 and the AUC for ADNP. **c** The modeling approach was validated using permutation tests, for which the performance measured as classification accuracy of the actual model is compared to control models in a cross-validation framework. For “random features”, fold-specific set of features of the same size as obtained by the LASSO-selection are chosen to train the model, and for “permuted labels” the modeling approaches are applied to shuffled group labels. The violin plots show the distribution of classification accuracies, for 10 repetitions and 100 permutations for the control models, and the *P* values indicate the median over the 10 repetitions of the exact *P* values obtained by permutation testing. **d** The bar graph depicts how often antibody features were selected by repeated LASSO-based selection. The color indicates the group in which the feature is enriched. The horizontal line shows the threshold of how often a feature needs to be chosen overall in order to be selected for the final set of minimal features. **e** A co-correlate network was constructed using Spearman rank correlations. Only correlations with |r| > 0.5 to at least one of the two LASSO-selected features, which are highlighted in gray, are shown.

Finally, we evaluated the second adjuvant cluster, including Alum (in the form of Al(OH)_3_) and AS04. Again, these adjuvants have different compositions, as AS04 contains MPL in addition to a different aluminum salt (i.e., AlPO_4_). Most features showed comparable levels for both adjuvants (Fig. 5a), and only a few features would be considered significantly different before correcting for multiple testing. To take into account the multivariate profile when comparing these adjuvants, we repeated the LASSO and PLS-DA modeling procedures. The model demonstrated partial separation (Fig. 5b, c) using LASSO-selected features. The top selected features included both the overall and the day 60 ADNP levels (Fig. 5d), marking a selection of neutrophil functions induced by AS04 (as well as by AS01/AS03; Fig. 3a), but not by Alum. Surprisingly, the two selected features were not strongly correlated with the other features, highlighting the specific axis of immunity leveraged by these two adjuvants (Fig. 5e). Interestingly, most of the features initially chosen by LASSO (Fig. 5d) related to antibody functions as opposed to simple titers, with only NK-cell-activating antibody levels being consistently higher in the Alum group. Overall, these results point towards qualitative differences between antibody profiles induced by the AS04-adjuvanted versus the Alum-adjuvanted vaccine (*Fendrix* and *EngerixB*, respectively; both GSK).

Thus, as our understanding of the importance of antibody Fc-effector functions in controlling different pathogens continues to evolve, the study presented here points to the potential utility of specific adjuvants in driving select antibody-effector profiles when rationally designing future vaccines.

## Discussion

The effectiveness of AS01_B_, AS01_E_, AS03, and AS04 in quantitatively enhancing immune responses to vaccine antigens has guided the wide deployment of licensed AS-containing vaccines, such as those against herpes zoster, malaria, influenza, and HPV^1,18,19,30^. We previously probed the adjuvanticities of these combination adjuvants and of Alum, for different arms of the immune response^23–26^. These analyses provided insights into the innate immune underpinnings that contribute to diverging adaptive response magnitudes and Fab properties induced by these adjuvants. Here, mirroring previously observed patterns^23–26^, the Fc functions induced by the second dose were distinct across the adjuvants, driven by 4 antibody features. The separation of the cluster formed by AS01_B_, AS01_E_, and AS03, from AS04 and Alum, was driven largely by antibody responses that were enriched in the AS01_B_/AS01_E_/AS03 cluster. Moreover, while responses induced by the latter cluster were uniformly detected after each immunization, responses in the AS04 and Alum groups did not emerge until after the antigenic challenge. Deeper analyses revealed comparable robustness across the AS01_B_, AS01_E_, and AS03 profiles, though with a trend for higher responses with AS01_B_. Moreover, granular analysis of the AS04/Alum profile revealed the presence of a unique signature for the AS04-induced humoral immune responses. Finally, the low-dose antigenic challenge given 10 months post last dose was at least as immunogenic with respect to antibody functionality as the adjuvanted doses, consistent with previous analyses of Fab-mediated humoral responses^26^. Thus, our findings suggest that, once the programming by the adjuvanted antigenic exposures is ‘hardwired’ into the memory B cells, it will dictate the encoded response features also after a non-adjuvanted boost or anamnestic response.

Systems serology offers a complementary high-throughput multivariate approach for deep antibody profiling, to explore differences in antibody profiles across vaccine strategies. While previous systems serology studies have noted quantitative differences in vaccine-induced isotypes and subclass titers across adjuvanted and non-adjuvanted vaccines^31^, the current study examined differences in the role of adjuvants in shaping Fc-receptor-binding profiles across a number of adjuvants. Importantly, low-affinity IgG Fc receptors are found on all immune cells, in different combinations. Because antibody:Fc-receptor interactions are low-affinity, multimerized antibodies (located in immune complexes) are involved in binding to diverse combinations of Fc receptors on innate immune cells at the site of infection. As a consequence, combinatorial, rather than univariate, differences likely shape Fc-effector functions more profoundly. Thus, while univariate analysis may provide unique insights into the impact of adjuvants on shaping individual components of the vaccine-induced humoral immune response, the multivariate profile, particularly in the context of Fc-effector functions, may provide critical insights into the inclusion of adjuvants that can induce the functions with the highest potential to contribute to the most potent antibody–pathogen responses. This knowledge is critical for rational vaccine development.

The minimal feature-set separating AS01/AS03 from AS04/Alum suggests that the dichotomy in antibody functionalities was driven by differences in the robustness of both the peak and the overall induced humoral responses. This divergence was detectable across several antibody-effector functions, including activation of phagocytosis by innate cells and of NK-cell-related responses. Specifically, the separation was based on peak titers of IgG_1_, which binds to all FcGRs^32^, longitudinal titers of both IgA_1_ (which correlated with FcAR-binding antibody levels) and IgM, and on persisting FcGRIIA-engaging responses (correlating with most FcGR-related responses). Not only did the data mirror the patterns previously observed in innate or humoral (total Ig, memory B cells, avidity) responses^23–26^, they also reflected the fact that the interferon (IFN)- and NK-cell-related blood transcriptional responses were uniquely detected with AS01 or AS03^24^. Thus, the separate innate profiles induced by AS01/AS03 or by AS04/Alum^23^ may also drive the antibody functionalities seen here for these clusters, though final conclusions are hampered by the limited sample size of our analyses. Both the gene signatures and innate responses shared by AS01 and AS03 were previously found to correlate statistically with the total Ig levels^23,24^, but whether they also correlate with functional antibody profiles remains to be determined. Overall, the data suggest that the robust, IFN-biased innate immunity and T/B-cell differentiation stimulated by AS01_B_, AS01_E_, or AS03 shaped both the Fab- and Fc-mediated humoral responses, although the mechanisms by which this could operate are not known.

The relationship between antibody functionality and protection was evaluated for the RTS,S/AS01_B_ malaria vaccine in adults, using systems serology tools^7^. As roughly half of these vaccinees were protected against subsequent malaria challenge, these data enabled linking a protective outcome with certain antibody functions. Many of these features overlapped with our current data for HBsAg/AS01_B_ (e.g., decreased IgM levels; increased levels of IgA_2_ and of FcGRIIIA-binding or cellular phagocytosis-/NK-cell-activating antibodies). The observation that the vaccine engendered both protective and nonprotective antibody profiles in a population is relevant, considering the differences in interindividual response variability detected previously between the four AS-adjuvanted HBsAg vaccines^23,24^. Understanding the molecular/immunological basis for this interindividual heterogeneity is thus of interest. Indeed, previous data revealed a marked variability in innate and transcriptional responses post-dose 2 among subjects receiving these HBsAg vaccines, with within-group heterogeneity progressively decreasing from AS04 to AS03, then AS01_E_, and then AS01_B_^23,24^. Thus, future studies linking antibody-effector function to vaccine-specific single-cell transcriptomics may provide mechanistic insights on how adjuvants differentially shape Fc-functional responses. Combining such future data with the individual transcriptional data and the current group-based data for these subjects may identify which innate signaling pathways lead to a functional response in a given individual. Finally, the high immunogenicity of the low-dose antigenic challenge, observed here for the Fc-functional responses in AS01/AS03 recipients and some AS04 recipients, was mirrored by the increased antibody avidity seen post-challenge in these subjects^26^. A proposed mechanism entails a preferential selection of the memory B-cell subsets producing highly functional antibodies, as dictated by the antigen-limiting milieu^33,34^. Thus, robust innate “imprinting” of memory B cells by an effective adjuvant may promote both Fab/Fc-functional recall responses.

The antigen-sparing, robust adjuvanticity of AS03 has been exploited in (pre)pandemic influenza vaccines against the A(H1N1)pdm09 and H5N1 strains, amongst others, and trivalent seasonal influenza vaccines for older adults^35–39^. By stimulating IFN-related gene expression and CD4^+^ T-cell responses, AS03 potentiated both quantitative (memory B-cell, hemagglutination inhibition/IgG_1,3_ levels) and qualitative (affinity, repertoire breadth) humoral response features for these vaccines^35,40–42^. Interestingly, Fc-mediated functions were also activated by H5N1 vaccines that were adjuvanted with MF59 (Novartis), though these responses lacked NK-cell and monocyte-phagocytic features^31^. Why these features were detected here for HBsAg/AS03 is unclear due to the many variables between these studies (e.g., antigen; population priming status; differences in constituents including α-tocopherol [present in AS03]). This may warrant further research. The broad efficacy across populations, and associated licensure statuses and safety databases of AS03-formulated influenza vaccines have informed the selection of AS03 for use in recombinant spike/receptor-binding domain protein SARS-CoV-2 candidate vaccines. The latter vaccines were immunogenic in preclinical and Phase 1/2 studies^20,21,43,44^, and various Phase 2/3 studies are underway. The rapidly accumulating (non)clinical data suggest that prerequisites of an efficacious SARS-CoV-2 vaccine with low immunopathological potential are robust responses of polyfunctional, T helper 1-biased CD4^+^ T cells, and of strongly neutralizing mucosal antibodies^45–48^. Both response types were detected following injection of the AS03-adjuvanted trimeric subunit vaccines in NHPs, which were then protected against subsequent viral challenge^43,44^. Emerging evidence also points to protective roles for IgA and Fc-functional antibodies^49–51^. Along with the experience gained so far with AS03^39,52,53^, the Fc-functional breadth of the antibody response observed here for HBsAg/AS03, supports the adjuvant selection for SARS-CoV-2 candidate vaccines. The use of a potent adjuvant for these vaccines is particularly relevant for older adults, due to age-related immunity impairment in this population^18^.

As for the transcriptional/innate responses, two doses of AS04 elicited a unique functional signature that differed vastly from the AS01/AS03 signatures, and subtly from the Alum signature. The data were consistent with the ranking of functional responses in the above-mentioned NHP study (i.e., MF59 > MPL + Alum > Alum)^22^. Similar trends were seen in the quantitative and qualitative (avidity) antibody responses to HBsAg^25,26^ or HPV^54,55^ vaccines adjuvanted with AS04 or Alum. Here, the divergence between the two adjuvants was rooted in increased neutrophil-mediated phagocytosis-related (ADNP) features for AS04. The collective effects are likely explained by increased innate signaling mediated by MPL–TLR4 engagement in AS04^56^. Compared with Alum-mediated effects, this increase can enhance neutrophil recruitment–apparent in the blood of these subjects^23^ and in murine lymph nodes^56^–possibly promoting antibody production, B-cell differentiation, and class switching^57,58^. However, the impact of neutrophil recruitment on the adjuvanted vaccine response is still unclear^59^. Finally, it is noted that some of the currently observed differences could be driven by the presence of non-responder participants in the AS04 group (Fig. 5).

In conclusion, AS01_B/E_ and AS03 have overall comparably strong abilities to modulate antibody isotypes, subclasses and Fc-binding profiles. These data can explain the consistent effectiveness of licensed vaccines containing these adjuvants, across an array of populations and vaccine antigens. Interestingly, AS04 was able to activate different humoral functionalities, providing it with a unique profile, which may contribute to the effectiveness of the AS04-adjuvanted HPV-16/18 vaccine^60^. Our findings can guide holistic strategies toward identifying optimal adjuvants for novel vaccines and indications and improving antigen-sparing immunization regimens.

The results highlight several avenues for future work. First, differences in interindividual response variability across these adjuvants^23–26^ suggest that baseline (epi)genetic differences between individuals define the composition of the humoral vaccine response. Functional profiling at the individual subject level could focus on the interaction between the innate pathways controlling the differences in functional antibody profiles across adjuvants and individuals, and the baseline interindividual variability. If indeed any relationships between innate immunity and antibody profile can be established, it is tempting to hypothesize that epigenetic changes induced by adjuvanted vaccines, as recently exemplified by H5N1/AS03^61^, could also affect the antibody profiles. Second, to trace back the cellular origin of the diverging functional profiles for the adjuvants, single-cell B-cell receptor (BCR) analysis can provide insight into the adjuvants’ capabilities to overrule pre-existing B-cell profiles. This can support immunization strategies for pathogen-primed individuals. BCR analysis can determine whether diverging antibody functionalities are harbored either by B-cell clones with different specificities, or by cells responding to dominant epitopes that dictate all detected functionalities. This consolidates our knowledge of interactions between Fc- and Fab-mediated functions. Third, new adjuvant development will be supported by deeper insights into the role of the adjuvants’ physicochemical properties—e.g., in the case of AS03, the structure of the oil-in-water emulsion, with the size of droplets being one among several parameters of interest^53^—and the ensuing differences in (bio)physical interactions with the antigen and with various types of innate immune cells. A last angle worth exploring is how to best exploit the robust functional antibody responses following the late antigenic challenge for the fine-tuning of recall vaccination regimens. This requires careful balancing of potential risks of a suboptimal protection of the target population versus the benefits of adjuvant/antigen sparing. Another application would be to include such challenge as a preparatory step, or even an alternative, for human challenge studies.

**Fig. 6:**
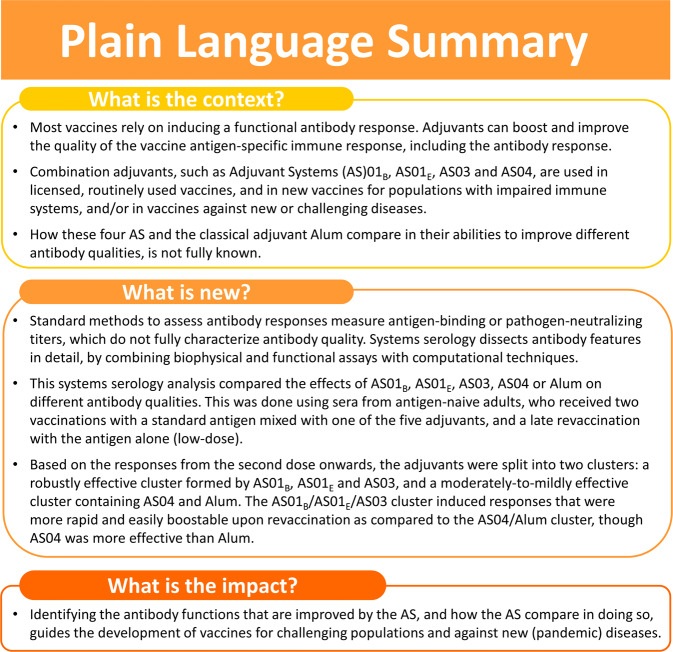
Plain language summary. Study overview and main implications described in a manner that is understandable by a non-specialist audience.

A plain language summary of the work presented here is provided in Fig. 6.

## Methods

### Study summary

This exploratory post-hoc analysis was conducted using serum samples from 18 to 45-year-old, HBV-naive male and female participants in a Phase II, randomized multicenter trial (NCT00805389)^23–26^. Subjects were immunized intramuscularly with 20 μg HBsAg adjuvanted with AS01_B_ (*n* = 15), AS01_E_ (*n* = 20), AS03 (*n* = 25), AS04 (*Fendrix*; *n* = 18), or Alum (*Engerix-B*; *n* = 21) on days 0 and 30. On day 360, the participants were revaccinated intramuscularly with a non-adjuvanted reduced-antigen (5 μg HBsAg) dose. The serum samples used for antibody profiling were collected on days 30, 60, 360, and 390.

Experimenters at Massachusetts General Hospital (MGH) were blinded as to the sample identity until all assay data had been collected. Assays performed at MGH using serum samples from the trial were deemed not human research following review by the MGH Institutional Review Board (IRB; protocol 2018P001039). In addition, human whole blood and buffy coats were collected at MGH from healthy donors who did not participate in the trial. The use of these internal samples as sources of uninfected primary neutrophils, monocytes, and NK cells was deemed not human research by the MGH IRB (protocols 2010P002121 and 2005P001218).

### Adjuvants

Each dose of AS01_B_ contained 50 μg MPL (3-*O*-desacyl-4′-monophosphoryl lipid A), 50 μg QS-21 (*Quillaja saponaria* Molina, fraction 21; licensed by GSK from Antigenics LLC., a wholly owned subsidiary of Agenus Inc., a Delaware, USA corporation), and liposome. Each dose of AS01_E_ contained 25 μg of MPL, 25 μg of QS-21, and liposome. Each dose of AS03 contained 11.86 mg DL-α-tocopherol and squalene in an oil-in-water emulsion. Each dose of AS04 contained 50 μg MPL adsorbed on aluminum salt (500 μg Al^3+^ in the form of AlPO_4_). Each dose of Alum contained 500 μg Al^3+^ in the form of Al(OH)_3_.

### Fluorescent primary and secondary antibodies

The following fluorescent antibodies were purchased from BD Biosciences: allophycocyanin (APC)-Cy7 anti-huCD14 (#557831, diluted at 1:100 in 5% BSA in PBS [PBSA]), phycoerythrin (PE)-Cy7 anti-huCD56 (#335791, diluted at 1:100 in PBSA), and BV421 anti-huMIP1β (#562900, diluted at 1:100 in PBSA). Additional fluorescent antibodies were purchased from BioLegend: Pacific Blue anti-huCD66b (#305112, diluted at 1:100 in PBSA), BV785 anti-huCD3 (#300472, diluted at 1:100 in PBSA), APC-Cy7 anti-huCD3 (#300426, diluted at 1:100 in PBSA), BV605 anti-huCD107a (#328634, diluted at 1:50 in PBSA), and PE anti-huIFNγ (#506507, diluted at 1:100 in PBSA). A fluorescein isothiocyanate (FITC)-conjugated, goat anti-guinea pig complement C3 polyclonal antibody was purchased from MP Biomedical (#0855385, diluted at 1:500 in PBSA). PE-conjugated secondary antibodies were purchased from Southern Biotech for the detection of total huIgG (#9040-09), huIgM (#9020-09), huIgA_1_ (#9130-09), huIgA_2_ (#9140-09), huIgG_1_ (#9052-09), huIgG_2_ (#9070-09), huIgG_3_ (#9210-09), and huIgG_4_ (#9200-09).

### Antigen coupling to fluorescent beads

Yellow-green (#F8823) and blue (#F8814) fluorescent 1 μm carboxylate-modified microspheres were purchased from Thermo Fisher. Magnetic 1 μm carboxylate-modified microspheres were purchased from Bangs Laboratories (#MFY0001). Magplex-C microspheres were purchased from Luminex Corp. The carboxylate-modified, 1 μm microspheres (9 × 10^8^) or Magplex-C microspheres (5 × 10^6^) were covalently coupled to 25 μg HBsAg (provided by GSK) using a two-step carbodiimide reaction. Beads were washed and resuspended in 100 mM NaH_2_PO_4_, pH 6.2, and activated by incubating with 500 μg Sulfo-NHS (N-hydroxysulfosuccinimide, Pierce, #A39269) and 500 μg EDC (1-ethyl-3-[3-dimethylaminopropyl] carbodiimide-HCL, Pierce, #A35391) for 30 min at room temperature. The beads were washed three times with coupling buffer (50 mM MES, pH 5.0), then incubated with protein antigen in 500 μl of coupling buffer for two hours at room temperature. The beads were washed three times with PBS-TBN (1XPBS (phosphate-buffered saline), 0.1% bovine serum albumin (BSA), 0.02% Tween-20, and 0.05% sodium azide, pH 7.4) and blocked with PBS-TBN for 30 min at room temperature. Beads were then washed three times with PBS, 0.05% Tween-20, and resuspended in storage buffer (1XPBS, 0.05% sodium azide).

### Antibody isotype and subclass analysis

The isotypes and subclasses of HBsAg-specific antibodies were quantified using a previously described method^62^. In this method, Magplex-C microspheres were coupled to HBsAg via carbodiimide crosslinking with Sulfo-NHS and EDC, as described above. These beads were then blocked with 5% BSA in PBS (PBSA) and added to black flat-bottom 384-well plates (Greiner Bio-One, #781906) so that each well contained 1500 HBsAg-coupled beads. Serum from test subjects was diluted in PBSA and co-incubated with the beads for two hours at room temperature on a plate shaker (800 rpm). The beads were then washed and incubated with a PE-conjugated antibody to detect total human IgG, huIgG_1_, huIgG_2_, huIgG_3_, huIgG_4_, huIgM, huIgA_1_, or huIgA_2_ for one hour at room temperature on a plate shaker (800 rpm). Antibodies were diluted and blocked in Luminex Assay buffer (1XPBS pH = 7.4, 0.1% w/v BSA, 0.05% Tween-20) using the following concentrations: total huIgG = 1:200, huIgG_1_ = 1:200, huIgG_2_ = 1:200, huIgG_3_ = 1:200, huIgG_4_ = 1:200, huIgM = 1:100, huIgA_1_ = 1:100, and huIgA_2_ = 1:100. The beads were then washed and resuspended in 40 µL of QSOL (IntelliCyt, Sartorius, # 91304). Fluorescence readouts were acquired on an Intellicyt iQue Screener PLUS flow cytometer (Intellicyt/Sartorius, #11811) and data was read and exported from iQue Forecyt V 10.0.8341 (Intellicyt/Sartorius, #60028). Results were reported as the median PE fluorescence intensity (MFI) and averaged across technical duplicates for each sample. All samples were tested at two dilutions to accurately capture IgG data at low (days 30 and 360) and high titer time points (days 60 and 390), while a single sample dilution was sufficient to capture data for the other isotypes and IgG subclasses.

### Fc-binding protein array

The binding of HBsAg-specific antibodies to human Fc receptors and complement C1q was measured using a previously described assay^63,64^. In this assay, avi-tagged FcGR2A(H), FcGR2A(R), FcGR2B, FcGR3A(V), FcGR3A(F), FcGR3B, FcRn, and FcAR proteins were custom produced and purified by the Duke Human Vaccine Institute Protein Production Facility. 100 µg of these proteins were then biotinylated with BirA ligase using a commercially available kit (Avidity, #BirA500). Purified human C1q protein (Sigma, #C1740) was biotinylated using EZ-Link Sulfo-NHS-LC-LC-Biotin (Pierce, #A35358) according to the manufacturer’s instructions. 16 µg of the biotinylated Fc domain-binding proteins were then incubated for 10 min with 4 µg of streptavidin-PE (Prozyme, #PJ31S) followed by 10 min with 5 μM D-biotin (Thermo Fisher, #B20656) to generate the assay detection reagents. Magplex-C microspheres (Luminex MFG, #MC12001-X, cataloged by region) were coupled to 25 µg HBsAg as described above, blocked with PBSA, and added to 384-well plates (Thermo Fisher, #460518) so that each well contained ≥1500 HBsAg-coupled beads. Serum from test subjects was diluted in PBSA (1:500 for IgG_1_, 1:100 for IgG_2_, 1:250 for IgG_3_, 1:100 for IgG_4_, 1:100 for IgM, 1:100 for IgA_1_, 1:100 for IgA_2_, and 1:1000 for all Fc receptors) and added to the beads, and incubated for two hours at room temperature on a plate shaker (800 rpm). The beads were then washed 3X using the 384-well HydroSpeed Plate Washer (Tecan, #30190112), incubated with one of the PE/FcR conjugates for 1 h at room temperature on a plate shaker (800 rpm), washed again, and acquired on an Intellicyt iQue Screener PLUS flow cytometer. Results were reported as the median PE fluorescence intensity, averaged across technical duplicates for each sample. For FcAR- and C1q-binding antibodies, a single sample dilution was sufficient to capture data for all samples in the study. For the other readouts, all samples were tested at two dilutions to accurately capture FcR-binding antibody data at low (days 30 and 360) and high titer time points (days 60 and 390).

### THP-1 monocyte phagocytosis assay

An assay for measuring antibody-dependent THP-1 monocyte phagocytosis was used as previously described^65^. In this assay, 1 μm yellow-green fluorescent beads (Thermo Fisher, #F8776) were coupled to HBsAg and blocked overnight with PBSA. The beads were then washed twice manually with PBSA, diluted to 1.8 × 10^8^ beads/ml, and 10 μl beads/well were added to a 96-well round-bottom microplate (Costar, #3799). Diluted serum from immunized subjects (10 μl/well) was added to the beads and incubated at 37 °C for 2 h, to allow the formation of immune complexes. Unbound antibodies were washed off manually, then 25,000 THP-1 cells/well (ATCC, #TIB-202) were added to the beads in 200 μl THP-1 medium (RPMI (Corning 15-040-CV) + 10% FBS + 55 μM β-mercaptoethanol) and incubated overnight at 37 °C. Cells were fixed and acquired on an Intellicyt iQue Screener PLUS flow cytometer. The phagocytic score for each sample was calculated as (% bead-positive cells) × (gMFI of bead-positive cells)/(10 × gMFI of first bead-positive peak), where gMFI refers to geometric mean fluorescence intensity, and results were reported as the mean phagocytic score of technical duplicates for each sample. All samples were tested at two dilutions to accurately measure phagocytosis at low (days 30 and 360) and high titer time points (days 60 and 390).

Future studies, aimed at examining downstream effects of antibodies on shaping myeloid activation, maturation, and cytokine secretion, could further highlight differences in adjuvant-mediated humoral immune programming^66^.

### Primary neutrophil phagocytosis assay

An assay for measuring ADNP has been described previously^67^. In this assay, 1 μm yellow-green fluorescent beads (Thermo Fisher, #F8776) were coupled to HBsAg and blocked with PBSA overnight at 4 °C. The beads were then washed twice manually with PBSA and diluted to 1.8 × 10^8^ beads/ml. HBsAg-coupled beads (10 μl/well) and diluted test sera (10 μl/well) were combined in a round-bottom 96-well plate, then incubated at 37 °C for 2 h. Primary leukocytes were isolated from freshly drawn whole blood (collected from healthy donors in anticoagulant citrate dextrose tubes) by treatment with ammonium-chloride-potassium (ACK) red blood cell lysis buffer (Thermo Fisher, #A1049201), then diluted in RPMI + 10% FBS media to 250,000 cells/ml. After immune complex formation, the beads were washed, combined with 50,000 primary leukocytes/well, and incubated for an hour at 37 °C. Cells were stained for surface CD66b (BD Biosciences #305112), CD14 (BD Biosciences, #557831), and CD3 (BD Biosciences, #558117), all diluted at 1:100 in PBSA, fixed with 4% paraformaldehyde (Santa Cruz Biotechnologies, #SC-281692), and acquired on an Intellicyt iQue Screener PLUS flow cytometer. Gates were drawn to identify singlet SSC^high^ CD66b^+^ CD14^−^ CD3^−^ cells, and phagocytic scores for each sample were calculated as (% bead-positive cells) × (gMFI of bead-positive cells)/(10 × gMFI of the first bead-positive peak). Samples were assayed in duplicate using primary neutrophils isolated from two donors, and results were reported as the mean phagocytic score for each sample. All samples were tested at two dilutions to accurately measure phagocytosis at both low (days 30 and 360) and high titer time points (days 60 and 390).

### Primary MoDC phagocytosis assay

Primary monocytes were isolated from healthy donor huPBMCs using CD14 positive-selection microbeads (Miltenyi, #130-050-201), then grown in vitro for 6 days in MoDC differentiation medium containing granulocyte-macrophage colony-stimulating factor (GM-CSF) and IL-4 (Miltenyi, #130-094-812). Red fluorescent 1-μm beads (Thermo Fisher, #F8775) were coupled to HBsAg, blocked with PBSA, and then washed and diluted to 1.8 × 10^8^ beads/ml. Beads and diluted test sera (10 μl each/well) were combined in round-bottom 96-well microplates and incubated at 37 °C for 2 h. The beads were then washed and incubated with 40,000 primary MoDCs/well in R-10 medium at 37 °C for 4 h, then fixed and acquired on an Intellicyt iQue Screener PLUS flow cytometer. The phagocytic score for each sample was calculated as (% bead-positive cells) × (gMFI of bead-positive cells)/(10 × gMFI of first bead-positive peak). Samples were assayed in duplicate using MoDCs isolated from two donors, and results were reported as the mean phagocytic score for each sample. All study samples were assayed at a single sample dilution.

### Complement deposition assay

An assay for measuring ADCD was used as previously described^68^. In this assay, 1 μm red fluorescent beads (Thermo Fisher, #F8775) were coupled to 25 µg of HBsAg, blocked with PBSA, then washed and diluted to 1.8 × 10^8^ beads/ml. HBsAg-coupled beads (10 μl/well) were combined with diluted test sera (10 μl/well) in a 96-well round-bottom microplate (Costar, #3799), then incubated at 37 °C for 2 h. Guinea pig complement (CedarLane, #CL4051) was diluted in gelatin veronal buffer containing calcium and magnesium (GVB + +, Boston Bioproducts, #IBB-300). The beads were washed manually with PBS and incubated with diluted complement for 20 min at 37 °C. The beads were then washed with 5 mM EDTA, stained with FITC-conjugated anti-complement C3 (MP Biomed, #855385) diluted 1:500 in PBSA, and acquired on an Intellicyt iQue Screener PLUS flow cytometer. Gates were drawn on the singlet, red fluorescent particles, and complement deposition was reported as the median fluorescence intensity (MFI) on the FITC channel, averaged across technical duplicates for each study sample. All samples were assayed at two dilutions to accurately measure complement deposition at low (days 30 and 360) and high titer time points (days 60 and 390).

### NK-cell activation assay

An assay for measuring ADNKA has been described previously^69^. In this assay, flat-bottom 96-well ELISA plates (Thermo Fisher, #439454) were coated with 30 µg of HBsAg diluted in PBS, then blocked with PBSA. Serum samples from test subjects were diluted in PBSA, added to the plates, and incubated for two hours at 37 °C. Primary human NK cells were purified from buffy coats from healthy donors using the RosetteSep human NK-cell enrichment cocktail (StemCell, #15065), then resuspended in RPMI + 10% FBS media containing 10 μg/ml brefeldin A (Sigma, #B7651), GolgiStop (BD Biosciences, #554724, diluted 1:10 in PBS), and fluorescent anti-CD107a (BD Biosciences, #555802, diluted 1:50 in PBSA). The ELISA plates were washed three times manually with PBS, then isolated NK cells (25,000/well) were added and incubated at 37 °C for 5 h. The cells were then stained for surface CD56 (BD Biosciences, #557747, diluted 1:50 in PBSA) and CD3 (BD Biosciences, #558117, diluted 1:50 in PBSA), permeabilized using Fix and Perm Cell Permeabilization Kit (Thermo Fisher, #GAS002S-100), stained with fluorescent antibodies to IFN-γ (ΒD Biosciences, #340449, diluted 1:100 in PBSA) and MIP-1β (ΒD Biosciences, #550078, diluted 1:100 in PBSA), fixed, and acquired on an Intellicyt iQue Screener PLUS flow cytometer. Gates were drawn on the singlet, CD56^+^/CD3^−^ cells, and results were reported as the percentages of these cells that expressed surface CD107a, intracellular MIP-1β, or intracellular IFN-γ. Samples were assayed in duplicate using NK cells isolated from two donors, and results were averaged for each sample. All study samples were assayed at a single sample dilution.

### Statistical analysis

All calculations were performed with R Studio software version 4.0.2 (Open Source). Measurements for antibody isotypes, subclasses and ADCD were log_10_-transformed. If multiple dilutions were generated, the dilutions used for day 60 and day 390 were used for all days in the analyses for the comparison of days. Significances were assessed using paired Wilcoxon tests, and corrected for multiple testing using the Benjamini–Hochberg procedure (R function “p.adjust”). For the heatmaps in Figs. 3a, 4a, and 5a, the data for each day were z-scored across the compared groups or clusters of groups, and the color of the tiles indicates the difference in average z-score. Significances were assessed using Mann–Whitney *U* tests. Adjusting the *P* values for multiple testing, no difference is significant with false discovery rate (FDR) < 0.05.

### Feature selection and classification of adjuvant

For the multivariate analysis, missing data for four individuals at day 30 (3 AS01_E_, 1 AS04), 1 individual at day 60 (Alum), and four individuals at day 390 (1 AS01_E_, 2 AS03 and 1 Alum) was imputed using k-nearest neighbor imputation employing the function “knnImputation” (with parameter *k* = 10) of the R package “DMwR”. For each time point, eight isotypes/subclasses, nine FcR-binding affinities and seven functional scores were measured. Measurements for antibody isotypes, subclasses, and ADCD were log_10_-transformed. From the functional features, a polyfunctionality score was calculated for each individual as the number of functional readouts (ADCD, ADCP, ADNP, ADDCP, ADNKA [CD107a, IFN-γ, MIP-1β]) that were above the median across all individuals. This yielded in total 25 antibody features per time point. To dissect differences in the responses to the adjuvants, we used each antibody feature at each time point and the overall AUC that combined the measurements (except polyfunctionality) for all time points, yielding a total of 124 features. To find the most discriminating features between adjuvant groups, we employed a LASSO-based selection procedure^70^. First, the measurements were z-scored to have mean 0 and standard deviation 1 across all vaccinees. Next, the function “cv.glmnet” of the R package “glmnet” with a binomial or multinomial distribution assumption was used to determine which features are important to discriminate the groups and, thus, have an estimated nonzero coefficient. This procedure was repeated ten times, and features were selected that had a nonzero coefficient in more than a pre-defined fraction of repetitions (0.9 for the comparison of all adjuvants, 0.9 for the merged subgroups, 0.1 for AS01_B_/AS01_E_/AS03, and 0.9 for AS04/Alum). The low threshold for the comparison of the non-separable adjuvants AS01_B_/AS01_E_/AS03 was required to ensure that features were chosen in the selection process (Supplemental Fig. 2B). The thresholds were also indicated in the corresponding bar graphs as horizontal lines. For the bar graphs this procedure was repeated 100 times. Using the LASSO-selected features, PLS-DA models (using the R package “ropls”) were built to discriminate the adjuvant groups. For the score plots, ellipses indicate the 75% confidence regions assuming a multivariate *t* distribution. For all shown models, either the *R*^2^ for the second component/latent variable (LV) was <0.01 or the Q^2^ < 0.05, and, thus, the component would not be included in the model and was only calculated for visualization purposes.

### Model validation

The modeling approach was assessed for robustness using tenfold cross-validation, for which the selection procedure and PLS-DA modeling was performed fold-specific. For each fold, the labels of this fold were predicted using the model trained on the remaining data, and classification accuracies were obtained by comparing the predicted labels for all data to the true labels after iterating through all tenfolds. Furthermore, permutation tests were used, in order to assess the significance of the modeling approach^71^. For this, two types of control models were generated: (1) “random features”, which selected fold-specific random feature sets of the same size as the features set selected by the actual modeling approach, and (2) “permuted labels”, for which the whole modeling approach was applied to data with shuffled group/adjuvant labels. This procedure was done using 100 permutations for each of 10 cross-validation replicates. The *P* values for the modeling approach were then obtained from the tail probability of the generated null distribution, i.e., the distribution of classification accuracies of the control models.

### Correlation analysis

Correlation heatmaps were generated using Spearman rank correlations. For the correlation networks, only significant (Benjamini–Hochberg-adjusted *P* value <0.05) correlations that were higher than a certain threshold, and to a selected feature, are shown.

### Reporting summary

Further information on research design is available in the Nature Research Reporting Summary linked to this article.

## Supplementary information

Supplemental Material

REPORTING SUMMARY

## Data Availability

GSK makes available anonymized individual participant data and associated documents from interventional clinical studies which evaluate medicines, upon approval of proposals submitted to www.clinicalstudydatarequest.com. To access data for other types of GSK-sponsored research, for study documents without patient-level data, and for clinical studies not listed, please submit an inquiry via the website. Study number: NCT00805389.

## Code availability

Full code used for the analyses presented in this manuscript is available upon request to the corresponding author.
