# Supplementary material for: Systems serology-based comparison of antibody effector functions induced by adjuvanted vaccines to guide vaccine design
Source: NPJ Vaccines. 2023 Mar 8;8:34. doi: 10.1038/s41541-023-00613-1 (PMC9992919; doi:10.1038/s41541-023-00613-1)
Supplement: Supplementary file 1 — Supplemental Material [file 41541_2023_613_MOESM1_ESM.pdf]

Supplemental Figure 1

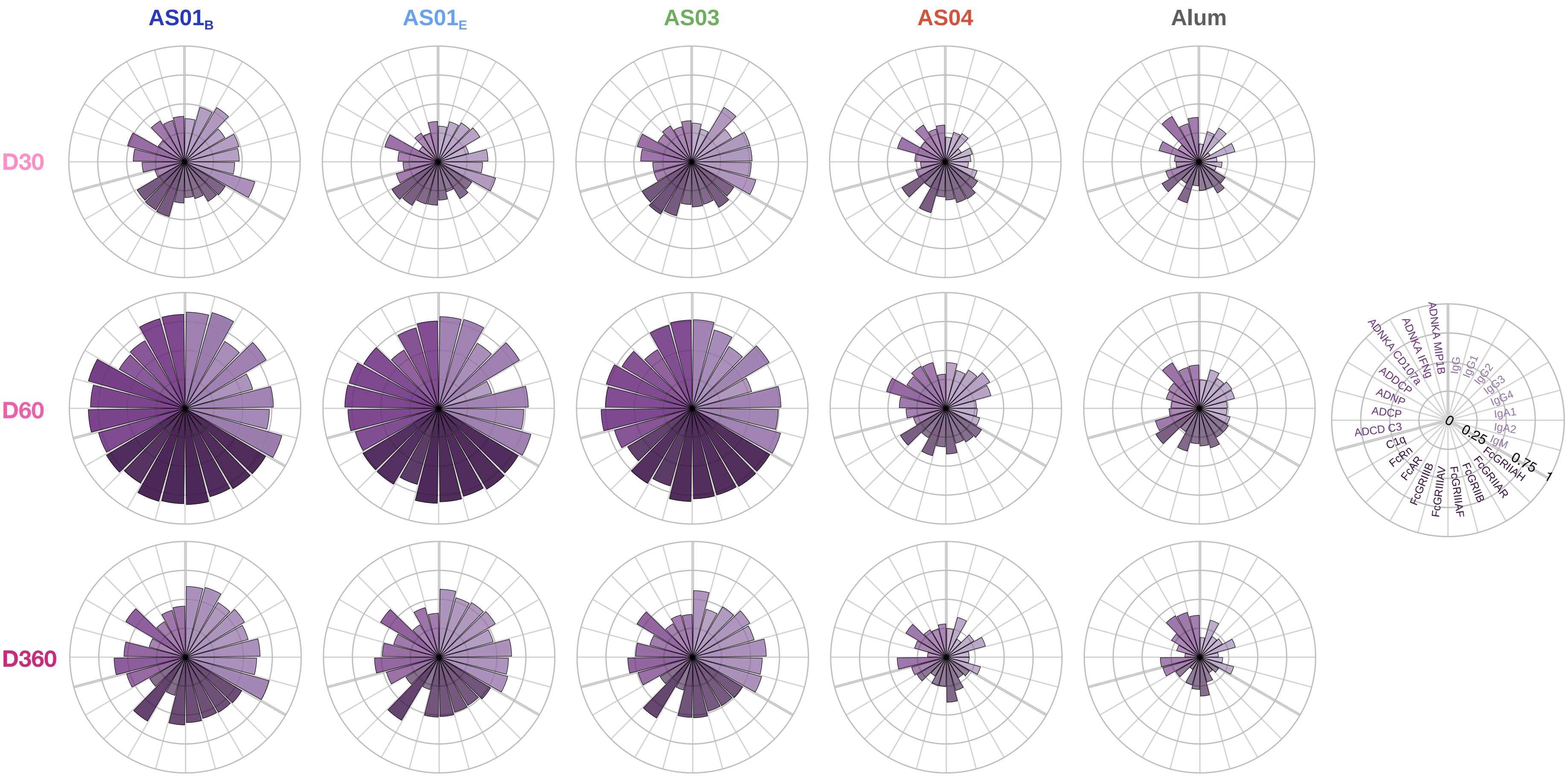

Supplemental Fig. 1: The polar plots depict the mean percentile of each antibody feature for each adjuvant group at day 30, day 60 and day 360. Percentile rank scores were determined for each antibody feature across all individuals, adjuvants and time points.

Supplemental Figure 2

a

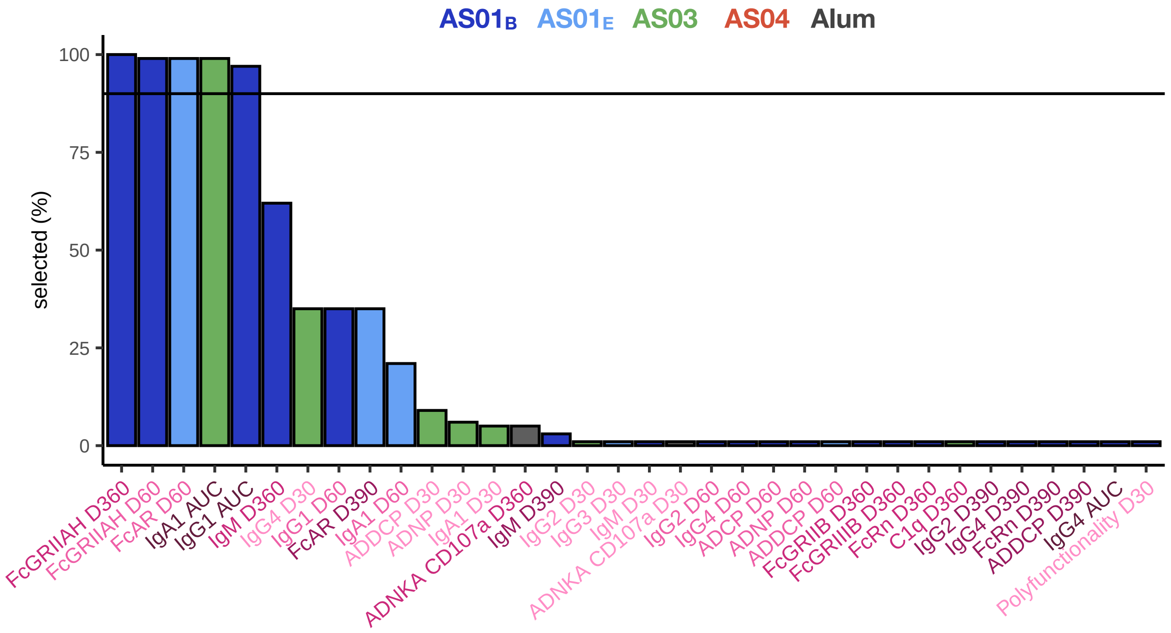

b

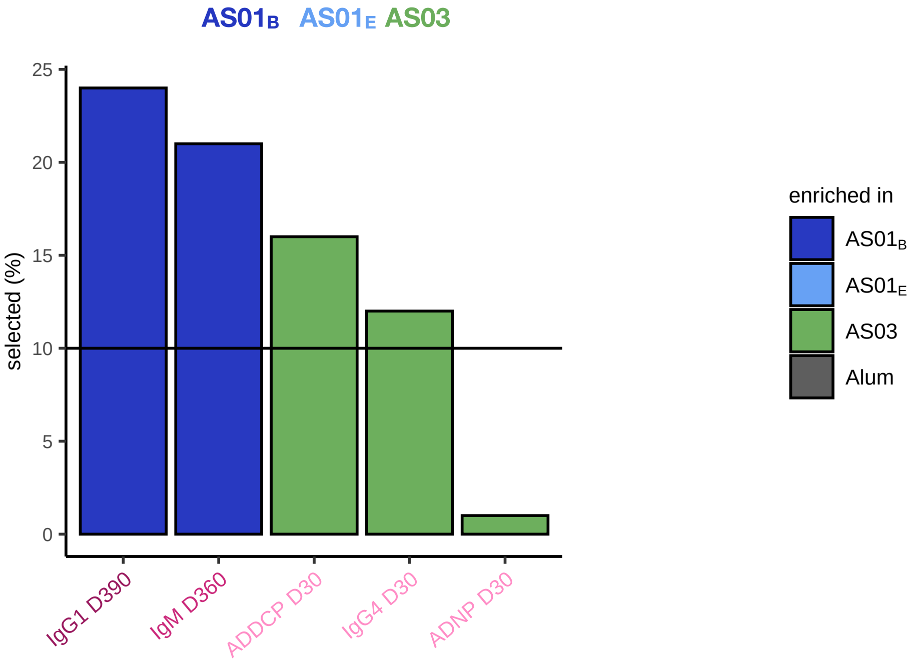

Supplemental Fig. 2: (a, b) The bar graphs depict how often antibody features were selected by repeated LASSO-based selection. The color indicates the group in which the feature is enriched. The horizontal line shows the threshold of how often a feature needs to be chosen overall in order to be selected for the final set of minimal features. The features correspond to the model comparing **(a)** all five adjuvants (corresponding to Fig. 1d) and **(b)** $AS01_B$, $AS01_E$, and AS03 (corresponding to Fig. 4b).

[illegible] Antibody features at different time points for each vaccine adjuvant group

Adjuvant	Feature	lineChart_D30		lineChart_D60		lineChart_D360		lineChart_D390	
		Z-score median	IQR	Z-score median	IQR	Z-score median	IQR	Z-score median	IQR
AS01$_B$	IgG	0.162	0.456	0.830	0.873	0.904	1.254	0.979	0.894
AS01$_B$	IgG1	0.010	1.240	1.362	0.789	1.011	2.231	1.246	0.734
AS01$_B$	IgG2	0.384	1.232	0.575	1.533	-0.035	1.741	0.412	1.136
AS01$_B$	IgG3	0.139	0.875	0.826	1.153	0.361	1.096	0.516	1.169
AS01$_B$	IgG4	-0.194	1.139	0.011	1.003	-0.154	0.779	-0.097	1.113
AS01$_B$	IgA1	0.269	1.370	0.562	0.712	0.805	1.244	0.474	0.973
AS01$_B$	IgA2	-0.081	1.254	0.504	0.904	0.639	1.374	0.905	0.738
AS01$_B$	IgM	0.670	1.659	0.507	0.715	0.681	0.712	0.733	0.613
AS01$_B$	FcGRIIAH	0.671	1.334	1.013	0.279	0.986	0.422	0.974	0.308
AS01$_B$	FcGRIIAR	-0.199	0.694	1.011	0.721	0.899	1.995	1.108	0.458
AS01$_B$	FcGRIIB	0.433	1.291	0.941	0.504	1.074	0.450	1.106	0.490
AS01$_B$	FcGRIIIAF	-0.144	0.740	0.966	1.241	1.161	0.821	1.207	1.464
AS01$_B$	FcGRIIIAV	-0.162	0.837	0.983	0.596	1.115	1.310	1.127	0.754
AS01$_B$	FcGRIIIB	-0.270	0.286	0.048	1.672	1.073	1.115	1.049	2.040
AS01$_B$	FcAR	0.096	1.953	0.748	0.734	0.838	1.642	0.749	1.081
AS01$_B$	FcRn	-0.158	0.048	0.125	1.733	0.702	1.865	1.291	2.074
AS01$_B$	C1q	-0.125	0.109	0.773	1.515	-0.207	0.091	0.969	1.179
AS01$_B$	ADCD C3	0.119	0.512	0.989	0.718	0.306	0.692	1.115	0.819
AS01$_B$	ADCP	0.249	0.673	0.872	1.569	0.660	0.951	0.826	0.576
AS01$_B$	ADNP	0.013	1.127	0.474	1.377	-0.322	0.432	0.206	0.954
AS01$_B$	ADDCP	-0.184	1.040	0.132	1.022	0.463	1.655	0.878	0.941
AS01$_B$	ADNKA CD107a	0.023	0.942	0.345	0.972	-0.196	1.546	-0.258	1.012
AS01$_B$	ADNKA IFNg	0.206	0.545	0.579	0.980	-0.178	0.259	0.724	1.594
AS01$_B$	ADNKA MIP1B	0.207	1.159	0.777	1.119	0.238	1.149	0.675	1.904
AS01$_E$	IgG	-0.104	0.460	0.612	1.309	0.286	0.883	0.526	0.891
AS01$_E$	IgG1	-0.172	0.670	0.669	1.044	0.116	1.396	0.539	1.198
AS01$_E$	IgG2	-0.413	0.693	0.005	1.904	0.299	1.277	0.159	1.409
AS01$_E$	IgG3	-0.086	1.643	0.788	0.911	0.013	1.176	0.427	1.431
AS01$_E$	IgG4	-0.520	0.276	-0.235	0.431	-0.222	0.317	-0.101	0.962
AS01$_E$	IgA1	-0.295	1.249	0.753	1.100	0.629	1.110	0.528	1.260
AS01$_E$	IgA2	-0.362	0.747	0.626	0.941	0.216	1.199	0.750	0.914
AS01$_E$	IgM	-0.082	1.133	0.517	1.204	0.010	1.335	-0.071	0.689
AS01$_E$	FcGRIIAH	0.033	1.257	0.925	0.627	0.731	0.434	0.761	0.503
AS01$_E$	FcGRIIAR	-0.281	0.436	0.935	0.958	-0.129	1.006	0.607	0.817
AS01$_E$	FcGRIIB	-0.229	1.357	0.838	1.080	0.775	0.714	0.726	0.745
AS01$_E$	FcGRIIIAF	-0.367	0.339	0.728	1.504	0.808	1.022	0.120	0.972
AS01$_E$	FcGRIIIAV	-0.165	0.387	0.924	1.162	0.700	1.311	0.567	0.768
AS01$_E$	FcGRIIIB	-0.293	0.244	-0.371	1.547	0.473	1.541	-0.294	1.343
AS01$_E$	FcAR	-0.158	1.544	0.839	0.844	0.707	1.515	0.800	1.004
AS01$_E$	FcRn	-0.164	0.114	-0.109	1.724	-0.418	1.191	-0.336	0.754
AS01$_E$	C1q	-0.089	0.059	0.429	1.902	-0.227	0.254	0.517	1.178
AS01$_E$	ADCD C3	-0.050	0.465	0.696	1.443	-0.099	0.720	0.655	0.950
AS01$_E$	ADCP	-0.127	0.711	0.883	1.230	0.608	0.858	0.589	1.263
AS01$_E$	ADNP	-0.373	0.770	0.435	1.368	-0.102	0.433	-0.216	0.697

$AS01_E$	ADDCP	-0.500	1.127	0.356	0.943	0.102	1.259	0.337	1.336
$AS01_E$	ADNKA CD107a	-0.372	0.818	-0.019	0.973	-0.336	0.968	-0.132	0.916
$AS01_E$	ADNKA IFNg	-0.219	0.459	0.101	1.719	-0.149	0.232	-0.373	0.768
$AS01_E$	ADNKA MIP1B	0.075	1.370	0.057	1.641	-0.212	0.790	-0.031	1.333
AS03	IgG	0.055	0.647	0.525	1.139	0.295	1.212	0.349	1.117
AS03	IgG1	-0.437	0.731	0.219	1.360	-0.624	1.202	0.246	1.707
AS03	IgG2	0.091	1.570	0.091	1.001	0.065	1.096	0.201	1.138
AS03	IgG3	-0.047	1.286	0.558	1.077	0.391	1.549	0.465	1.079
AS03	IgG4	0.043	1.468	-0.176	0.366	-0.123	0.540	-0.291	0.769
AS03	IgA1	0.804	1.371	0.514	1.206	0.537	1.290	0.378	1.013
AS03	IgA2	0.087	1.820	0.477	1.394	0.443	1.437	0.537	0.700
AS03	IgM	0.269	1.253	0.401	1.170	0.300	0.682	0.085	0.853
AS03	FcGRIIAH	0.404	1.173	0.859	0.580	0.815	0.422	0.794	0.712
AS03	FcGRIIAR	-0.048	1.116	0.863	0.665	0.030	1.783	0.741	1.265
AS03	FcGRIIB	0.059	1.147	0.831	0.696	0.883	0.509	0.694	1.006
AS03	FcGRIIIAF	-0.341	0.782	0.305	1.265	0.749	1.146	-0.131	1.846
AS03	FcGRIIIAV	-0.043	0.840	0.796	0.711	0.646	1.376	0.608	1.351
AS03	FcGRIIIB	-0.257	0.273	-0.346	1.756	0.593	1.436	-0.279	1.633
AS03	FcAR	0.555	1.773	0.461	1.237	0.446	1.453	0.441	1.055
AS03	FcRn	-0.157	0.121	-0.438	1.085	-0.277	1.875	-0.125	1.298
AS03	C1q	-0.155	0.104	0.193	1.695	-0.198	0.384	0.181	1.379
AS03	ADCD C3	-0.133	0.506	0.786	1.461	-0.208	0.806	0.824	1.870
AS03	ADCP	0.054	1.105	-0.061	1.359	0.466	1.291	0.650	1.375
AS03	ADNP	-0.094	1.122	0.082	1.089	-0.025	0.829	-0.297	1.302
AS03	ADDCP	0.088	1.079	0.268	1.319	0.052	1.352	0.532	0.942
AS03	ADNKA CD107a	-0.152	0.899	-0.002	1.082	-0.215	1.267	-0.527	0.545
AS03	ADNKA IFNg	-0.226	0.505	0.111	1.365	-0.219	0.210	-0.195	1.786
AS03	ADNKA MIP1B	-0.040	1.027	0.306	1.286	-0.319	0.876	-0.048	1.452
AS04	IgG	-0.391	0.266	-0.952	0.094	-0.813	0.202	-0.970	0.807
AS04	IgG1	-0.416	0.278	-0.990	0.186	-0.626	0.099	-0.968	0.534
AS04	IgG2	-0.595	0.908	-0.577	0.775	-0.704	0.329	-0.735	0.663
AS04	IgG3	-0.644	0.205	-0.933	0.558	-0.904	0.543	-0.944	0.712
AS04	IgG4	-0.556	0.241	-0.339	0.151	-0.357	0.134	-0.523	1.264
AS04	IgA1	-0.711	0.336	-0.988	0.294	-0.904	0.217	-0.921	1.500
AS04	IgA2	-0.610	0.177	-0.945	0.186	-0.798	0.125	-1.104	1.111
AS04	IgM	-0.580	0.467	-1.014	0.225	-0.943	0.429	-0.692	0.508
AS04	FcGRIIAH	-0.727	0.738	-1.094	0.286	-1.111	0.727	-1.162	1.152
AS04	FcGRIIAR	-0.384	0.340	-1.018	0.139	-0.676	0.125	-1.054	0.557
AS04	FcGRIIB	-0.616	0.197	-1.020	0.062	-1.171	0.195	-1.076	0.427
AS04	FcGRIIIAF	-0.446	0.100	-0.868	0.030	-1.044	0.081	-0.848	0.067
AS04	FcGRIIIAV	-0.468	0.256	-1.033	0.060	-0.924	0.127	-1.059	0.426
AS04	FcGRIIIB	-0.299	0.076	-0.552	0.021	-0.908	0.038	-0.700	0.027
AS04	FcAR	-0.722	0.247	-1.016	0.287	-0.897	0.131	-1.160	1.592
AS04	FcRn	-0.150	0.122	-0.564	0.056	-0.634	0.077	-0.649	0.075
AS04	C1q	-0.135	0.127	-0.792	0.030	-0.291	0.114	-0.903	0.037
AS04	ADCD C3	-0.109	0.447	-0.960	0.048	-0.432	0.304	-0.958	0.159
AS04	ADCP	-0.620	0.549	-0.911	0.123	-0.956	0.145	-0.929	0.584

AS04	ADNP	-0.386	0.784	-0.575	0.303	-0.409	0.193	-0.568	0.146
AS04	ADDCP	-0.110	1.500	-0.676	0.357	-0.545	0.648	-0.681	0.994
AS04	ADNKA CD107a	-0.101	0.951	-0.520	0.460	-0.253	0.817	-0.393	1.834
AS04	ADNKA IFNg	-0.098	0.387	-0.694	0.083	-0.289	0.222	-0.675	1.018
AS04	ADNKA MIP1B	-0.178	0.665	-0.842	0.185	-0.319	0.698	-0.593	0.603
Alum	IgG	-0.577	0.251	-1.026	0.055	-0.862	0.136	-1.097	0.100
Alum	IgG1	-0.436	0.310	-0.971	0.169	-0.636	0.210	-1.022	0.372
Alum	IgG2	-0.514	0.751	-0.806	0.423	-0.696	0.333	-0.684	0.497
Alum	IgG3	-0.689	0.193	-1.229	0.209	-0.918	0.266	-1.091	0.384
Alum	IgG4	-0.384	0.532	-0.412	0.236	-0.345	0.203	-0.593	0.227
Alum	IgA1	-0.829	0.282	-1.050	0.101	-0.973	0.127	-1.199	0.624
Alum	IgA2	-0.641	0.209	-0.983	0.088	-0.789	0.093	-1.216	0.134
Alum	IgM	-0.789	0.176	-1.078	0.166	-0.962	0.514	-0.936	0.243
Alum	FcGRIIAH	-0.598	0.765	-1.120	0.209	-1.272	0.492	-1.250	0.071
Alum	FcGRIIAR	-0.352	0.357	-1.034	0.097	-0.700	0.086	-1.100	0.054
Alum	FcGRIIB	-0.725	0.255	-1.009	0.030	-1.257	0.215	-1.118	0.037
Alum	FcGRIIIAF	-0.454	0.081	-0.876	0.031	-1.051	0.055	-0.844	0.021
Alum	FcGRIIIAV	-0.458	0.202	-1.031	0.036	-0.967	0.125	-1.080	0.049
Alum	FcGRIIIB	-0.292	0.128	-0.554	0.024	-0.926	0.063	-0.702	0.018
Alum	FcAR	-0.754	0.309	-1.096	0.094	-0.942	0.072	-1.257	0.188
Alum	FcRn	-0.189	0.066	-0.562	0.065	-0.608	0.067	-0.675	0.042
Alum	C1q	-0.144	0.140	-0.777	0.026	-0.285	0.143	-0.919	0.015
Alum	ADCD C3	-0.150	0.350	-0.981	0.089	-0.556	0.377	-0.967	0.042
Alum	ADCP	-0.670	0.346	-1.011	0.049	-0.897	0.255	-1.155	0.219
Alum	ADNP	-0.475	0.592	-0.795	0.138	-0.385	0.128	-0.527	0.109
Alum	ADDCP	-0.399	0.869	-0.810	0.314	-0.878	0.550	-1.072	0.467
Alum	ADNKA CD107a	-0.038	0.895	-0.221	0.808	-0.133	1.923	-0.376	1.452
Alum	ADNKA IFNG	-0.024	0.394	-0.696	0.211	-0.242	0.300	-0.577	0.543
Alum	ADNK MIP1B	-0.199	1.047	-0.764	0.265	-0.111	1.007	-0.771	0.628

Notes: D, Day; IQR, interquartile range
