# Supplementary material for: Systems serology-based comparison of antibody effector functions induced by adjuvanted vaccines to guide vaccine design
Source: NPJ Vaccines. 2023 Mar 8;8:34. doi: 10.1038/s41541-023-00613-1 (PMC9992919; doi:10.1038/s41541-023-00613-1)
Supplement: Supplementary file 2 — REPORTING SUMMARY [file 41541_2023_613_MOESM2_ESM.pdf]

nature portfolio

Corresponding author(s): Wivine Burny

Last updated by author(s): Apr 8, 2022

Reporting Summary

Nature Portfolio wishes to improve the reproducibility of the work that we publish. This form provides structure for consistency and transparency in reporting. For further information on Nature Portfolio policies, see our Editorial Policies and the Editorial Policy Checklist.

Statistics

For all statistical analyses, confirm that the following items are present in the figure legend, table legend, main text, or Methods section.

n/a	Confirmed	
☐	☒	The exact sample size (*n*) for each experimental group/condition, given as a discrete number and unit of measurement
☐	☒	A statement on whether measurements were taken from distinct samples or whether the same sample was measured repeatedly
☐	☒	The statistical test(s) used AND whether they are one- or two-sided<br>*Only common tests should be described solely by name; describe more complex techniques in the Methods section.*
☐	☒	A description of all covariates tested
☐	☒	A description of any assumptions or corrections, such as tests of normality and adjustment for multiple comparisons
☐	☒	A full description of the statistical parameters including central tendency (e.g. means) or other basic estimates (e.g. regression coefficient) AND variation (e.g. standard deviation) or associated estimates of uncertainty (e.g. confidence intervals)
☐	☒	For null hypothesis testing, the test statistic (e.g. *F*, *t*, *r*) with confidence intervals, effect sizes, degrees of freedom and *P* value noted<br>*Give P values as exact values whenever suitable.*
☒	☐	For Bayesian analysis, information on the choice of priors and Markov chain Monte Carlo settings
☐	☒	For hierarchical and complex designs, identification of the appropriate level for tests and full reporting of outcomes
☐	☒	Estimates of effect sizes (e.g. Cohen's *d*, Pearson's *r*), indicating how they were calculated

Our web collection on statistics for biologists contains articles on many of the points above.

Software and code

Policy information about availability of computer code

Data collection	Full code used for the analyses presented in this manuscript is available upon request to the corresponding author.
Data analysis	Full code used for the analyses presented in this manuscript is available upon request to the corresponding author.

For manuscripts utilizing custom algorithms or software that are central to the research but not yet described in published literature, software must be made available to editors and reviewers. We strongly encourage code deposition in a community repository (e.g. GitHub). See the Nature Portfolio guidelines for submitting code & software for further information.

Data

Policy information about availability of data

All manuscripts must include a data availability statement. This statement should provide the following information, where applicable:

- Accession codes, unique identifiers, or web links for publicly available datasets
- A description of any restrictions on data availability
- For clinical datasets or third party data, please ensure that the statement adheres to our policy

GSK makes available anonymized individual participant data and associated documents from interventional clinical studies which evaluate medicines, upon approval of proposals submitted to www.clinicalstudydatarequest.com. To access data for other types of GSK sponsored research, for study documents without patient-level data and for clinical studies not listed, please submit an enquiry via the website (ClinicalTrials.gov identifier: NCT00805389).

Field-specific reporting

Please select the one below that is the best fit for your research. If you are not sure, read the appropriate sections before making your selection.

[X] Life sciences   [ ] Behavioural & social sciences   [ ] Ecological, evolutionary & environmental sciences

For a reference copy of the document with all sections, see nature.com/documents/nr-reporting-summary-flat.pdf

Life sciences study design

All studies must disclose on these points even when the disclosure is negative.

Sample size

This is an exploratory post-hoc analysis conducted using serum samples from 18-45 year old, HBV-naïve male and female participants in a Phase II, randomized multicenter trial (NCT00805389) (1–4). Subjects were immunized intramuscularly with 20 g HBsAg adjuvanted with AS01B (n = 15), AS01E (n = 20), AS03 (n = 25), AS04 (Fendrix; n = 18), or Alum (Engerix-B; n = 21) on days 0 and 30. On day 360, they were revaccinated intramuscularly with a non-adjuvanted reduced-antigen (5 g HBsAg) dose. The serum samples used for antibody profiling were collected on days 30, 60, 360, and 390.

References:
1- Burny et al. Front. Immunol. 8, 943 (2017).
2- De Mot et al. Sci Transl Med 12, eaay8618 (2020).
3- Leroux-Roels et al. Clin Immunol 169, 16-27 (2016).
4- Budroni et al. NPJ Vaccines 6, 78 (2021).

Data exclusions

N/A

Replication

N/A

Randomization

This is an exploratory post-hoc analysis conducted using serum samples from 18-45 year old, HBV-naïve male and female participants in a Phase II, randomized multicenter trial (NCT00805389) (1–4). Subjects were immunized intramuscularly with 20 g HBsAg adjuvanted with AS01B (n = 15), AS01E (n = 20), AS03 (n = 25), AS04 (Fendrix; n = 18), or Alum (Engerix-B; n = 21) on days 0 and 30. On day 360, they were revaccinated intramuscularly with a non-adjuvanted reduced-antigen (5 g HBsAg) dose. The serum samples used for antibody profiling were collected on days 30, 60, 360, and 390.

References:
1- Burny et al. Front. Immunol. 8, 943 (2017).
2- De Mot et al. Sci Transl Med 12, eaay8618 (2020).
3- Leroux-Roels et al. Clin Immunol 169, 16-27 (2016).
4- Budroni et al. NPJ Vaccines 6, 78 (2021).

Blinding

The observer-blind, randomized, controlled trial (ClinicalTrials.gov identifier: NCT00805389) was conducted from December 2008 to July 2011. Last collected data in 2019.

Reporting for specific materials, systems and methods

We require information from authors about some types of materials, experimental systems and methods used in many studies. Here, indicate whether each material, system or method listed is relevant to your study. If you are not sure if a list item applies to your research, read the appropriate section before selecting a response.

Materials & experimental systems

n/a	Involved in the study	
[ ]	[X]	Antibodies
[X]	[ ]	Eukaryotic cell lines
[X]	[ ]	Palaeontology and archaeology
[X]	[ ]	Animals and other organisms
[X]	[ ]	Human research participants
[ ]	[X]	Clinical data
[X]	[ ]	Dual use research of concern

Methods

n/a	Involved in the study	
[X]	[ ]	ChIP-seq
[ ]	[X]	Flow cytometry
[X]	[ ]	MRI-based neuroimaging

Antibodies

Antibodies used

BD Biosciences: allophycocyanin (APC)-Cy7 anti-huCD14 (#557831), phycoerythrin (PE)-Cy7 anti-huCD56 (#335791), and BV421 anti-huMIP1B(#562900).
BioLegend: Pacific Blue anti-huCD66b (#305112), BV785 anti-huCD3 (#300472), APC-Cy7 anti-huCD3 (#300426), BV605 anti-

huCD107a (#328634), and PE anti-huIFNg (#506507). A fluorescein isothiocyanate (FITC)-conjugated, goat anti-guinea pig complement C3 polyclonal antibody was purchased from MP Biomedical (#0855385). PE-conjugated secondary antibodies were purchased from Southern Biotech for the detection of total huIgG (#9040-09), huIgM (#9020-09), huIgA1 (#9130-09), huIgA2 (#9140-09), huIgG1 (#9052-09), huIgG2 (#9070-09), huIgG3 (#9210-09), and huIgG4 (#9200-09).

Validation: N/A

Clinical data

Policy information about clinical studies

All manuscripts should comply with the ICMJE guidelines for publication of clinical research and a completed CONSORT checklist must be included with all submissions.

Clinical trial registration	ClinicalTrials.gov identifier: NCT00805389
Study protocol	GSK makes available anonymized individual participant data and associated documents from interventional clinical studies which evaluate medicines, upon approval of proposals submitted to www.clinicalstudydatarequest.com. To access data for other types of GSK sponsored research, for study documents without patient-level data and for clinical studies not listed, please submit an enquiry via the website (ClinicalTrials.gov identifier: NCT00805389).
Data collection	The observer-blind, randomized, controlled trial (ClinicalTrials.gov identifier: NCT00805389) was conducted from December 2008 to July 2011. Last collected data in 2019.
Outcomes	N/A

Flow Cytometry

Plots

Confirm that:

- [ ] The axis labels state the marker and fluorochrome used (e.g. CD4-FITC).
- [ ] The axis scales are clearly visible. Include numbers along axes only for bottom left plot of group (a 'group' is an analysis of identical markers).
- [ ] All plots are contour plots with outliers or pseudocolor plots.
- [x] A numerical value for number of cells or percentage (with statistics) is provided.

Methodology

Sample preparation	Described in Cell 2015 Nov 5;163(4):988-98.
Instrument	Described in Cell 2015 Nov 5;163(4):988-98.
Software	Described in Cell 2015 Nov 5;163(4):988-98.
Cell population abundance	Described in Cell 2015 Nov 5;163(4):988-98.
Gating strategy	Described in Cell 2015 Nov 5;163(4):988-98.

- [ ] Tick this box to confirm that a figure exemplifying the gating strategy is provided in the Supplementary Information.
